# Interaction between ischemia-reperfusion injury and intestinal microecology in organ transplantation and its therapeutic prospects

**DOI:** 10.3389/fimmu.2024.1495394

**Published:** 2024-12-06

**Authors:** Yong-qi Lian, Peng-fei Li, Yan Guo, Yan-lin Tao, Ya-nan Liu, Zhao-yu Liang, Shu-fen Zhu

**Affiliations:** ^1^ Department of Critical Care Medicine, Affiliated Hospital of Inner Mongolia Medical University, Hohhot, Inner Mongolia Autonomous Region, China; ^2^ Department of Orthopaedics, Inner Mongolia Autonomous Region People’s Hospital, Hohhot, Inner Mongolia Autonomous Region, China; ^3^ Pathology Department, Inner Mongolia Autonomous Region People’s Hospital, Hohhot, Inner Mongolia Autonomous Region, China; ^4^ Department of Surgery ICU, Affiliated Hospital of Inner Mongolia Medical University, Hohhot, Inner Mongolia Autonomous Region, China; ^5^ Physical Examination Center, Affiliated Hospital of Inner Mongolia Medical University, Hohhot, Inner Mongolia Autonomous Region, China

**Keywords:** organ transplantation, ischemia-reperfusion injury, intestinal microecology, intestinal barrier, microbial therapy

## Abstract

Organ transplantation is a vital intervention for end-stage organ failure; however, ischemia-reperfusion injury is a complication of transplantation, affecting the prognosis and survival of transplant recipients. As a complex ecosystem, recent research has highlighted the role of the intestinal microecology in transplantation, revealing its significant interplay with ischemia-reperfusion injury. This review explores the interaction between ischemia-reperfusion injury and intestinal microecology, with a special focus on how ischemia-reperfusion injury affects intestinal microecology and how these microecological changes contribute to complications after organ transplantation, such as infection and rejection. Based on a comprehensive analysis of current research advances, this study proposes potential strategies to improve transplant outcomes, offering guidance for future research and clinical practice.

## 1 Introduction

Organ transplantation is an effective treatment option for end-stage organ failure. However, due to the inevitable ischemia-reperfusion injury in the process of donor organ acquisition, preservation, and transplantation as well as the function and survival of transplanted organs face challenges (1). Ischemia-reperfusion injury (IRI) is caused by cell hypoxia and the accumulation of metabolites during ischemia, followed by oxidative stress and inflammation during reperfusion in the donor organ, which not only affects the primary function of the transplanted organ but may also lead to long-term complications (2). Intestinal microecology is closely related to the occurrence and development of various diseases, including metabolic, immune, and infectious diseases (3). In recent years, increasing evidence has shown that the intestinal microecology plays an important regulatory role in solid organ transplantation. Therefore, understanding and regulating the impact of IRI on intestinal microecology has become an important research focus, aimed at improving the transplant success rates and patient survival quality. Starting from the mechanism of action of IRI in organ transplantation and intestinal microecology, this paper will deeply explore the interaction between them and analyze the main research progress and application prospects in this field.

## 2 Mechanisms of IRI

IRI is a serious pathological condition that poses a major challenge during organ transplantation, resulting in substantial cellular damage and death (4). It is a major cause of primary graft dysfunction, a delayed graft function, chronic graft dysfunction, graft rejection, and other post-transplant complications and contributes to increased morbidity and mortality among transplant recipients (5).

### 2.1 Pathophysiological changes during ischemia

**Figure 1:**
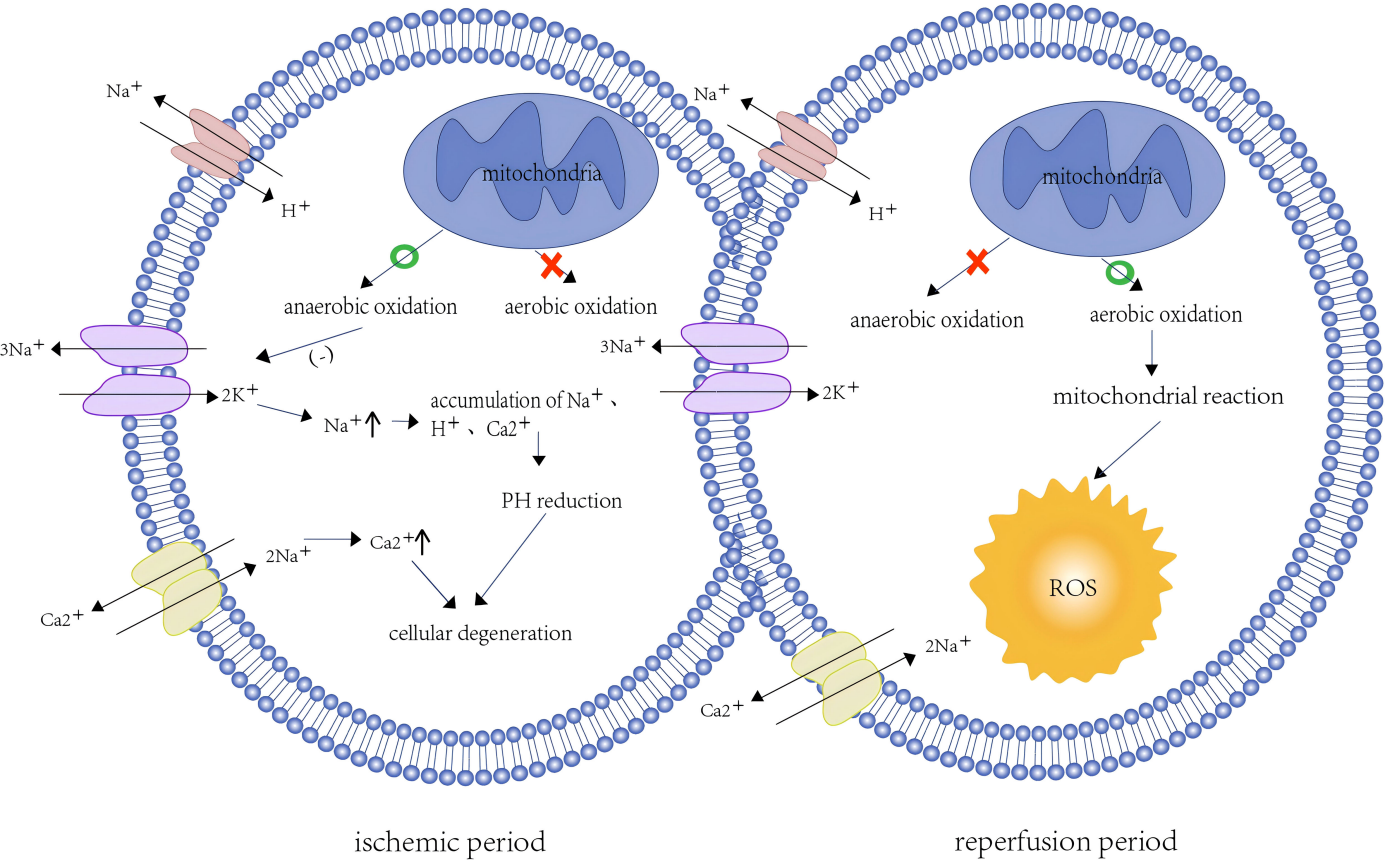
Intracellular changes during ischemia and reperfusion.

At the cellular level, ischemia leads to hypoxia, which disrupts cellular metabolism and reduces cellular bioenergetics by inhibiting the function of the mitochondrial electron transport chain (6). Low oxygen levels deplete ATP in the mitochondria and shift to anaerobic metabolism, which impairs the function of energy-dependent sodium-potassium pumps at the cell membrane (7). Reduced Na^+^ -K^+^ pump activity leads to intracellular Na^+^ accumulation and further inhibits Na^+^-H^+^ pump activity (8). Disruption of ionic homeostasis and maintenance of the membrane potential lead to intracellular Ca^2+^ overload through inhibition of the Na^+^-Ca^2+^ pump (9). The hypertonic state of cells occurs due to elevated intracellular Na^+^, H^+^, and Ca^2+^ concentrations, resulting in water influx into the cytoplasm to maintain osmotic balance, leading to cellular edema (10). The accumulation of H^+^ in cells and the production of lactic acid by glycolysis can reduce the cellular PH, leading to metabolic acidosis and conformational changes in proteins (1) (**Figure 1**). In addition, Ca^2+^ overload can lead to cell damage via excessive production of reactive oxygen species (ROS). ROS accumulation impairs protein folding in the endoplasmic reticulum and activates a range of protein complexes, such as mitogen-activated protein kinase, calcium/calmodulin-dependent protein kinase, protein kinase C, and receptor-interacting protein kinase, which trigger a pro-inflammatory cascade (11). Studies have shown that in the process of ischemia, succinate accumulation leads to ROS accumulation, and pyruvate dehydrogenase kinase 4, a mitochondrial enzyme, can mitigate IRI-induced kidney injury by inhibiting ROS generation (12).

### 2.2 Pathophysiological changes during reperfusion

Reperfusion refers to the process of restoring blood supply. Although this process provides much-needed oxygen and nutrients, it may exacerbate ischemic injury due to the presence of other pathological processes, such as increased production of ROS, excessive inflammatory response, and programmed cell death (13). Reintroduction of excess oxygen increases ROS production, which disrupts the antioxidant defense system of the cell and results in oxidative stress on the cell membrane (14) (**Figure 1**). This imbalance between the overproduction of ROS and the cell’s antioxidant capacity is termed oxidative stress.

In a study of mouse heart transplantation, knocking down the mediators of oxidative stress induced excessive activation of ROS and further aggravated the degree of cardiac tissue damage (4). Excessive ROS not only promotes inflammation but also contributes to endothelial cell injury. Oxidative stress caused by ROS induces the release of pro-inflammatory cytokines through the activation of nuclear factor-κB, whereas endothelial cell activation induces endothelial-leukocyte adhesion and endothelial-platelet aggregation, which destroys cellular structure, eventually leading to cell death and aggravating ischemic injury (15). This process stimulates the innate immune response, which in turn enhances the activation of the adaptive immune response and promotes graft allogeneic identification.

IRI significantly affects the prognosis of organ transplantation via a series of complex pathophysiological changes. Regardless of whether it is a metabolic disorder and cell damage during the ischemia phase or oxidative stress and inflammatory response during the reperfusion phase, IRI has profound effects on cells and tissues, potentially leading to organ dysfunction. The next focus will be on how this damage further exacerbates the occurrence of complications after transplantation by disturbing intestinal microecology.

## 3 Effects of IRI on intestinal microecology

The intestinal microecology is composed of intestinal flora, intestinal epithelial cells, and the intestinal mucosal immune system 9 (16). Under normal circumstances, the quantity and distribution of intestinal microorganisms are relatively constant, and the microbial flora is relatively balanced and stable. However, diseases, immunity, stress, and other factors may affect the quantity, activity, or location of intestinal flora, resulting in an imbalance in intestinal flora and affecting the function of other distant organs (17). Intestinal microecology not only maintains intestinal homeostasis through competition and cooperative relationships but also protects host health through multiple intestinal barrier mechanisms. During organ transplantation, the inflammatory response and tissue damage caused by IRI can directly impair the intestinal barrier, disrupt the structure and function of the intestinal microbial community, and affect the clinical outcome after transplantation.

### 3.1 Mechanical barrier failure

**Figure 2:**
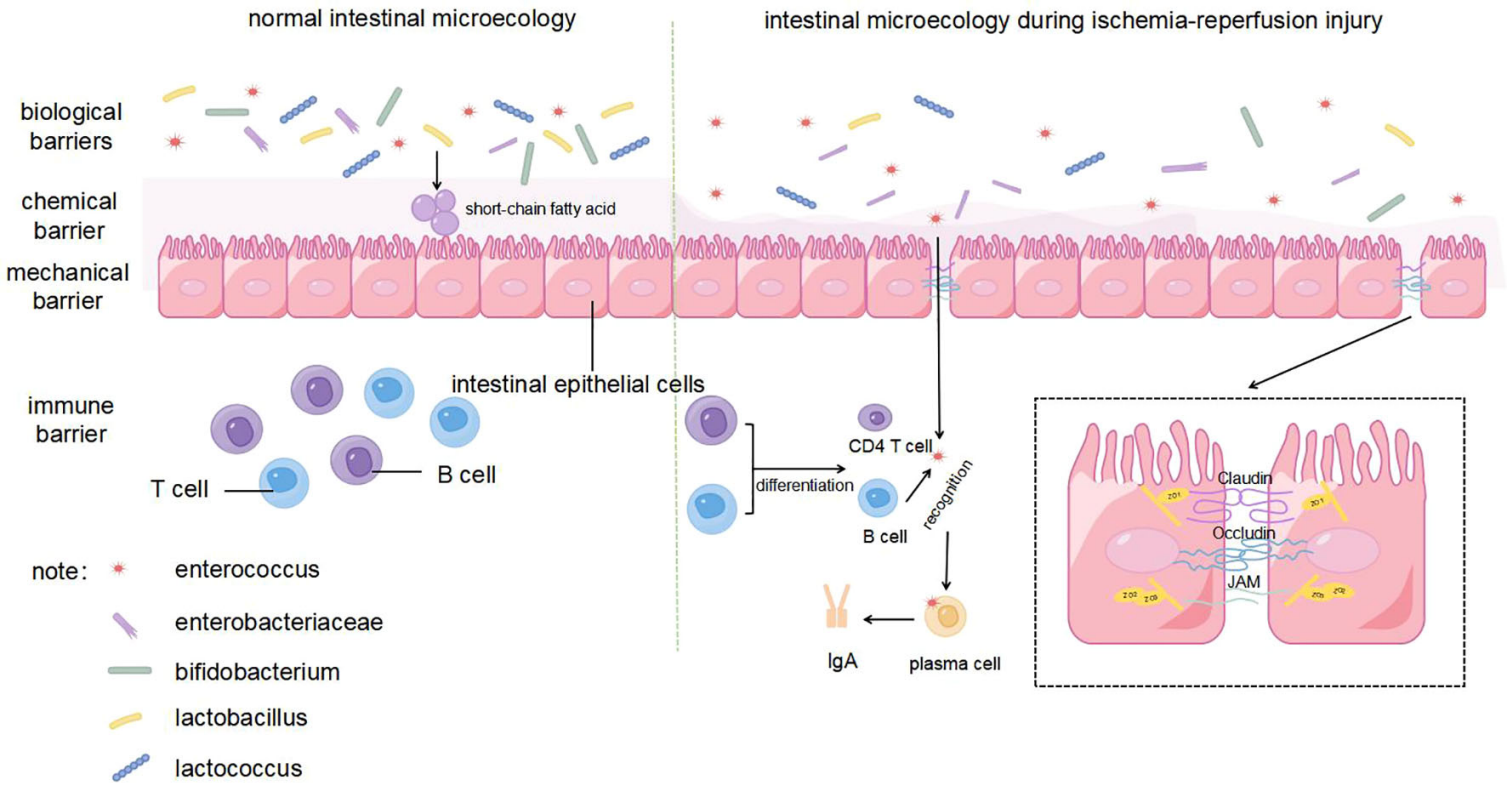
Intestinal microecological changes in normal intestine and ischemia-reperfusion injury.

The intestinal mechanical barrier is composed of intestinal epithelial cells and tight junctions, which are the first lines of defense to prevent the invasion of pathogenic microorganisms and toxins (18). Tight junctions are composed of membrane proteins, such as claudin, occludin, and junctional adhesion molecules, along with peripheral cytoplasmic proteins, such as zonula occludens (ZO), whose expression is inversely correlated with intestinal permeability (19) (**Figure 2**). During liver transplantation, intestinal blood flow is slowed due to clamping of the inferior vena cava and portal vein, and extensive intestinal mucosa is damaged by ischemia and hypoxia (20). The results of orthotopic liver transplantation male rats study showed that indicators representing an impaired intestinal barrier function (including endotoxin, diamine oxidase, and D-lactate levels) were significantly increased during IRI. At the same time, IRI after liver transplantation leads to the downregulation of Occludin and ZO-1 protein expression and increases epithelial cellular gaps in the rat intestine, which increases the risk of translocation of toxins and microorganisms through the paracellular pathway (21). Further studies have shown that intestinal epithelial cells are susceptible to the effects of IRI and then undergo apoptosis during transplantation. The expression of caspase-3 and apoptosis of intestinal epithelial cells increase significantly within 8 h of reperfusion after transplantation, indicating that the early stage of reperfusion is a critical period for intestinal barrier dysfunction (22). This phenomenon has also been verified in a kidney IRI model (23). In addition, the high level of endotoxins in the serum induces a host inflammatory response, which further exacerbates intestinal mucosal permeability (24).

### 3.2 Chemical barrier damage

The intestinal chemical barrier is mainly composed of a mucus layer secreted by intestinal mucosal epithelial cells, which can neutralize the acidic environment, prevent adhesion of pathogenic bacteria, and inhibit their growth (25) (**Figure 2**). The structure of the intestinal mucus layer is significantly altered during IRI, which may be related to ROS accumulation and inflammatory responses in the host (26). IRI stimulates the inflammatory response and oxidative stress by activating neutrophils, which release inflammatory cytokines and free radicals (27). Heme oxygenase (HO) is a specialized enzyme that degrades heme and is assembled from biliverdin, carbon monoxide, and free iron (28). HO-1 is normally expressed in the mucosal layer of the gastrointestinal tract and protects against stress-related tissue damage (29). In the intestinal transplantation and liver transplantation mouse model, compared with the control group, pretreatment with HO-1 or biliverdin can significantly inhibit leukocyte aggregation, thereby reducing the expression of proinflammatory cytokines and chemokines and ultimately diminishing systemic inflammation and oxidative stress (26, 30).

Regarding oxidative stress induced by IRI, researchers have found that glutamine and hydrogen can effectively reduce the oxidative stress response and even alleviate other organ complications in recipients by increasing endogenous cellular antioxidants (31). Hydrogen can play an antioxidant role by selectively neutralizing hydroxyl radicals. Because of its high diffusivity, it can reach mitochondria, which produce a large amount of ROS during IRI, and nuclear subcellular compartments, where ROS accumulation causes DNA damage (32). In addition, insufficient ATP synthesis caused by hypoxia reduces the frequency of ciliary oscillation, slows down intestinal peristalsis, and inhibits intestinal self-cleaning, which may cause accumulation of pathogenic bacteria in the intestine (33). These factors collectively contribute to impairment of the intestinal chemical barrier during organ transplantation, which in turn may trigger bacterial translocation and systemic inflammatory responses.

### 3.3 Immune barrier weakening

The immune barrier is an important part of the intestinal microecology and the body’s immune defense function, which protects the host from pathogenic antigen attack through both humoral and cellular immunity (34). Secretory immunoglobulin A (sIgA), the main immunoglobulin in intestinal secretions, is produced by lymphocytes and plasma cells throughout the intestinal mucosa and is a major defense mechanism against pathogen invasion by binding and neutralizing pathogens and preventing them from adhering to the surface of the intestinal mucosa (35) (**Figure 2**). Both the response to pathogens under normal conditions, such as harmless food antigens or commensal bacteria, and the induction of tolerance are the dual functions of sIgA in maintaining intestinal mucosal homeostasis. This process can be achieved by directly identifying the receptor binding domain of the pathogen to prevent the pathogen from interacting with epithelial cells and altering bacterial viability or pathogenicity (36). Specifically, sIgA promotes the health of intestinal epithelial cells and supports the expression of tight junction proteins, thereby maintaining the integrity of the intestinal barrier. In addition, sIgA may also indirectly enhance host immune defense by affecting the composition of gut microbiota. In the study by Lin et al., PBS-pretreated infected mice treated with probiotics exhibited higher sIgA levels and showed a more similar gut microbiota to healthy mice, as shown by a decrease in Proteobacteria and an increase in Bacteroidobacteria, along with a significant decrease in intestinal permeability and LPS levels. This suggests that sIgA can further effectively reduce the incidence of intestinal barrier damage by controlling intestinal microbiota homeostasis (37). Ischemic preconditioning before organ transplantation can effectively reduce fecal sIgA levels (38). In the mouse intestinal ischaemia/reperfusion model, IgA mRNA expression in the intestinal mucosa, sIgA concentration in the lavage fluid, and the percentage of bacteria coated with IgA in the feces of the injury group was lower than that of the sham-operated group 2 h after reperfusion, indicating that intestinal IRI not only reduced sIgA production but also impaired the bacterial binding ability of sIgA after reperfusion (39). These alterations set the stage for subsequent pathogen penetration into the intestinal barrier, facilitating bacterial translocation.

### 3.4 Biological barrier

The biological barrier is composed of a bacterial membrane layer formed by the attachment of deep intestinal microorganisms, such as Bifidobacterium and Lactobacillus, to the intestinal mucosa, which can maintain the balance of intestinal flora and inhibit the invasion and proliferation of pathogenic bacteria (**Figure 2**). Intestinal bacteria play a crucial role in forming biological barriers, hindering the entry of harmful bacteria and promoting the growth and maturation of the immune system by producing various enzymes (40). For example, some genera of Firmicutes can penetrate the mucus layer, which can stimulate intestinal epithelial cells to produce large amounts of antimicrobial proteins and limit the colonization of pathogenic microorganisms in the gut (41). In contrast, IRI can lead to a significant decrease in the diversity of the intestinal microbiota, manifested as a decrease in beneficial bacteria and an increase in harmful bacteria. For example, multiple liver transplantation studies have found that IRI leads to a significant decrease in beneficial bacteria, such as Lactobacillus and Bifidobacterium, while opportunistic pathogens, such as Enterococcus and Enterobacteriaceae, are increased (42). Simultaneously, the inflammatory response and oxidative stress caused by IRI also damage the intestinal mechanical, chemical, and immune barriers, thereby promoting bacterial colonization and translocation and further increasing the risk of enterogenous infection (39). For example, increased intestinal permeability, bacterial translocation, and endotoxemia can be clearly observed in a liver ischemia-reperfusion model (43).

In addition, researchers have found that an increase in Enterobacteriaceae and a decrease in Lactobacillus and Ruminococcus are markers of kidney IRI-induced ecological dysregulation and are associated with a decline in short-chain fatty acid (SCFA) levels (44). SCFAs are the main products of dietary fiber fermentation by intestinal flora, which play a crucial role in maintaining the intestinal barrier function, regulating immune responses, and preventing inflammation (45). Additional supplementation with SCFAs can significantly protect intestinal villi from IRI (46).

In conclusion, disruption of any intestinal defense barrier during organ transplant IRI can lead to intestinal microecological disorders. Once these intestinal defense barriers fail, the intestinal microecological balance is disrupted, causing intestinal damage and potentially triggering or exacerbating a series of post-transplantation complications. Understanding and intervening in these injury mechanisms are important for improving the success rate of organ transplantation and the prognosis of patients after transplantation.

## 4 Influence of intestinal microecology on complications after transplantation

The destruction of tight junction proteins and the inflammatory response caused by IRI can trigger intestinal flora imbalance, which increases the risk of infection, acute rejection, and graft death after transplantation.

### 4.1 Early postoperative infection

As one of the most common complications of solid organ transplantation, infection can significantly increase morbidity and mortality among patients (47) (**Table 1**). In 2018, Haak et al. indicated that a lack of butyrate-producing bacteria in the fecal microbiota was associated with an increased susceptibility to respiratory infections in allogeneic hematopoietic stem cell transplant recipients, at the same time, an abundance of butyrate producing bacteria >1% was associated with a 5-fold reduction in the development of future lower respiratory tract viral infections (48). Based on this, Lee et al. conducted a study involving 168 kidney transplant recipients and showed that the relative abundance of fecal butyrate-producing intestinal bacteria in the stool was >1% had a significantly lower risk of respiratory viral infections and influenza at 6 months, 1 year, and 2 years after transplantation than those with lower levels of these bacterias. The study also noted that high levels of butyrate-producing gut bacteria were associated with a reduced risk of cytomegalovirus viremia one year after kidney transplantation (49). Simultaneously, a decreased abundance of Firmicutes and *Faecalibacterium prausnitzii* and an increased proportion of Bacteroidetes and Proteobacteria were found in the intestines of kidney transplant recipients, which was closely related to the occurrence of infection after transplantation (52). Due to the anatomical connectivity of portal circulation, the liver is constantly exposed to bacterial products of intestinal microbiome origin, and Kato et al. found that loss of intestinal flora diversity is highly associated with the occurrence of bloodstream infections after liver transplantation (50). Antimicrobial therapy, which is commonly used to prevent or treat infections after transplantation, can lead to the colonization of multidrug-resistant pathogens, such as *Clostridium difficile*, further exacerbating ecological dysbiosis. Bruminhent et al. showed that *Clostridium difficile* infection (CDI) is an independent risk factor for mortality in the heart transplant population and is more common in patients undergoing retransplantation than the general population. Studies have shown that CDI has the highest frequency within one month of heart transplantation (53). Similarly, in the liver transplant population, colonization of multi-drug-resistant strains was significantly associated with increased levels of Enterococcus and Klebsiella and decreased levels of Bacteroides and Lachnospira in the gut microbiota (51). In addition, the authors suggested that the reduced alpha diversity of the intestinal microbiota before transplantation appears to be a marker of colonization by carbapenem-resistant Enterobacteriaceae following liver transplantation (51).

**Table 1:** Evidence of the association between intestinal microecology and infection in transplant recipients.

Subject of study	Changes in intestinal flora	Outcomes	Reference
360 patients receiving allogeneic hematopoietic stem cell transplants	The abundance of bacteria that produce butyrate decreases.	Within 6 months after transplantation, 41.4% of patients developed lower respiratory tract infection, the top three viruses were adenovirus, respiratory syncytial virus and human metapneumovirus.	(48)
168 kidney transplant patients	The abundance of butyrate-producing bacteria was altered.	Compared with the low abundance group, the high abundance group had a lower risk of respiratory virus infection at 6 months, 1 year and 2 years after transplantation, and a lower risk of cytomegalovirus infection within 1 year after transplantation.	(49)
38 liver transplant patients	The α diversity of bacterial flora was decreased.	During hospitalization, 8 patients (21.0%) had bloodstream infection, including 1 case of multi-species infection.	(50)
177 liver transplant patients	The genera Enterococcus and Klebsiella increased, and the levels of Bacteroides and Lachnospira decreased	The change of microbiota after transplantation is more likely to be infected with multi-drug-resistant bacteria, and the reduction of α diversity of intestinal flora before transplantation is a marker of carbapenem-resistant Enterobacteriaceae colonization.	(51)

### 4.2 Acute rejection

Acute rejection remains the leading cause of graft dysfunction after organ transplantation (54). Previous studies suggested that postoperative intestinal bacterial translocation plays a key role in the development of immune rejection (**Table 2**). Lei et al. showed that both germ-free and antibiotic-pre-treated mice exhibited a significant reduction in alloimmune responses and increased the graft survival after skin grafting. However, when these germ-free mice were transplanted with intestinal bacteria from conventional mice, skin graft rejection was accelerated, indicating that intestinal bacteria have an important impact on the process of rejection (55). Similarly, Wang et al. found that patients with decreased abundances of Firmicutes and Bacteroidetes and increased abundances of Proteobacteria, Actinobacteria, Lactobacillus, Fusobacteria, and Faecalibacterium in the intestinal flora after kidney transplantation were more likely to have graft rejection than those with a more balanced microbial composition or those who maintained higher levels of Firmicutes and Bacteroidetes (56). This was further supported by Kato et al., who reported that liver transplant recipients with rejection had a significant increase in *Bacteroidaceae*, *Enterobacteriaceae*, *Streptococcaceae*, and *Bifidobacteriaceae*, along with a significant decrease in *Enterococcaceae*, *Lactobacillaceae*, *Clostridiaceae*, *Ruminococcaceae*, and *Peptostreptococcaceae* in their intestines compared to healthy recipients (50). In animal models, mice with reduced levels of Enterococcaceae and Lactobacillaceae and increased levels of Clostridiaceae in the gut showed higher rejection (60). Similar phenomena have been observed in other organ transplants. For example, heart transplant mice treated with endogenous Bifidobacterium pseudolongum derived from the feces of pregnant mice showed lower rejection than those treated with conventional antibiotics or untreated controls (57). Using a multi-omics analysis, Wu et al. discovered that a species of the genus Bacteroides significantly reduced by 75% when it increased the acute rejection of lung transplantation (58). In patients undergoing small intestine transplantation, an increased Proteobacteria/Firmicutes ratio can be identified as a sensitive and specific indicator of rejection (59).

**Table 2:** Evidence of the association between intestinal microecology and acute rejection in transplant recipients.

Subject of study	Changes in intestinal flora	Outcomes	Reference
Skin transplantation mice	An increase in Lactobacillus in feces and a decrease in Clostridium in feces and skin.	Skin graft rejection was accelerated in germ-free mice receiving regular mouse feces	(55)
53 kidney transplant patients	Firmicutes and Bacteroidetes decreased, while that of Proteobacteria, Actinobacteria, Lactobacillus, Fusobacteria and Faecalibacterium increased.	Accelerated graft rejection, and Clostridium is a potential marker to distinguish rejection.	(56)
38 liver transplant patients	Bacteroidaceae, Enterobacteriaceae, Streptococcaceae, and Bifidobacteriaceae increased while that of Enterococcaceae, Lactobacillaceae, Clostridiaceae, Ruminococcaceae, and Peptostreptococcaceae decreased.	Acute rejection is associated with loss of gut microbial diversity.	(50)
Mice were pretreated with endogenous Bifidobacterium pseudolongum derived from the feces of pregnant mice	Bifidobacterium and Akkermansia increased after 2 weeks of gavage	The stability of the allograft was maintained.	(57)
82 lung transplant patients	Bacteroides increased.	Enrichment of monomorphic bacteroides modulates severe allograft dysfunction.	(58)
19 small intestine transplant patients	Streptococcaceae, Enterococcaceae, and Lactobacillaceae decreased and Enterobacteriaceae increased.	An increased Proteobacteria/Firmicutes ratio is a potential diagnostic marker for graft rejection	(59)

### 4.3 Graft survival rate

Recent studies have established a strong link between the intestinal microecology and improved graft survival outcomes after transplantation. First, Peled et al. demonstrated that a pattern of microbiota disruption, characterized by loss of diversity and dominance of a single taxon, was observed in allogeneic hematopoietic stem cell transplantation and then proposed an association between lower intestinal diversity and the risk of transplantation-related death, as well as death due to graft-versus-host disease (61). Specifically, a higher abundance of *Gammaproteobacteria*, including *Enterobacteriaceae*, was associated with higher mortality, whereas a higher abundance of Lachnospiraceae and Actinomycetaceae was associated with a better prognosis (62). Swarte et al. conducted a shotgun metagenomics analysis to investigate the impact of microbial dysbiosis on recipient survival following liver and kidney transplantation. Their study revealed that as the severity of microbial dysbiosis increased, the overall survival period of recipients significantly decreased. Specifically, by comparing the Shannon diversity index and Aitchison distance, they found that for every unit decrease in microbial diversity at the time of liver transplantation, the overall risk of mortality increased by 45%. Additionally, a smaller Aitchison distance was associated with a lower likelihood of mortality after liver transplantation, indicating a close relationship between microbial community similarity and recipient prognosis. This finding was also applicable to kidney transplantation (63). In addition, Willner et al. Found that the emergence of new Pseudomonas species after transplantation was associated with the most severe complications of lung transplantation (64). Based on this research, several specific taxa, including *Atopobium*, *Coprobacillus*, *Megamonas*, *Subdoligranulum*, and *Enterococcaceae*, were found to be significantly related to the risk of mortality following liver transplantation (63). In basic experiments, the use of mice from different suppliers confirmed that Alistipes can significantly affect the survival time of skin grafts (65). The intestinal microecology plays an important role in the development of complications after transplantation. Changes in intestinal microecology caused by ischemia-reperfusion injury not only increase the risk of early postoperative infection but are also closely related to acute rejection and graft mortality. A deeper understanding of how intestinal microecology impacts post-transplant complications is helpful in exploring new treatment strategies, thereby improving the success rate and long-term prognosis of patients after transplantation.

## 5 Application prospects of microbial therapy

In recent years, as our understanding of organ transplantation-related injuries has deepened, microbial therapy has emerged as a promising therapeutic strategy for preventing IRI (**Table 3**). This approach leverages manipulation of the gut microbiota to potentially mitigate the adverse effects of IRI, offering a novel strategy to improve outcomes in organ transplantation. Previous studies have shown that the recovery of intestinal barrier damage after IRI is closely related to the timing of intervention. According to the study of Kato et al., intestinal microecological intervention in the early stage after liver transplantation (within 72 hours after surgery) can significantly reduce the incidence of postoperative infection (52).

**Table 3:** Evidence of the association between microbial therapy and organ transplantation.

Subject of study	Treatment method	Outcomes	Reference
Mice	Probiotic preparations containing Blautia products were administered by gavage.	Improve vancomycin-resistant enterococcal infections.	(66)
66 liver transplant patients	Oral or nasogastric compound probiotic preparations consisting of *Pediacoccus pentosaceus, Leuconostoc mesenteroides, Lactobacillus paracasei*, and *Lactobacillus plantarum*.	It can effectively reduce the incidence of bacterial nosocomial infection after transplantation.	(67)
55 liver transplant patients	4 strains of probiotic preparations	The infection rate was significantly reduced in 30 days and 90 days after operation, and the liver function index was also reduced.	(68)
56 kidney transplant patients	Oral prebiotic supplements	It reduced the infection rate and improved the gastrointestinal symptoms after transplantation	(69)
94 solid organ transplant patients	FMT	Clostridium difficile infection was effectively reduced.	(70)
10 patients with Clostridium difficile infection	FMT	Clostridium difficile was transplanted by increasing the abundance of Firmicutes and Bacteroidetes.	(71)
3 lung transplant patients infected with multidrug-resistant bacteria	BT	2 patients were discharged after significant improvement in clinical symptoms, and no treatment-related adverse events occurred.	(72)

### 5.1 Probiotics and prebiotics

The International Scientific Society for Probiotics and Prebiotics defines probiotics as “living microorganisms that, when given in sufficient quantities, can exert beneficial effects on the health of the host” (73). The beneficial effects of probiotics are mainly achieved through the following mechanisms: inhibition of intestinal epithelial cell apoptosis, stimulation of mucus secretion, downregulation of pro-inflammatory mediators, upregulation of anti-inflammatory responses, interference with pathogen adhesion and invasion, and activation of innate and adaptive immune responses (74).

Most studies on the application of probiotics in organ transplantation have focused on animal models. Studies have shown that supplementation with probiotics containing Blautia products can reverse vancomycin-resistant Enterococcus infection in germ-free mice (69). Similar results have also been observed in clinical studies, in which the incidence of bacterial infections after liver and kidney transplantation was significantly reduced in patients treated with *Lactobacillus plantarum* (67, 75). The study by Grat et al. further confirmed that post-transplant infection rates were significantly reduced in liver transplant recipients supplemented with probiotics, along with improvements in biochemical markers of allograft function, such as bilirubin concentration and transaminase activity (68). The role of prebiotics in organ transplant complications is as important as that of a food source that promotes the growth of beneficial gut microbes. In kidney transplant recipients with gastrointestinal symptoms, significant suppression of infection and gastrointestinal symptoms was observed after seven weeks of treatment with prebiotic powder suspension (69).

### 5.2 Fecal microbial transplantation

**Figure 3:**
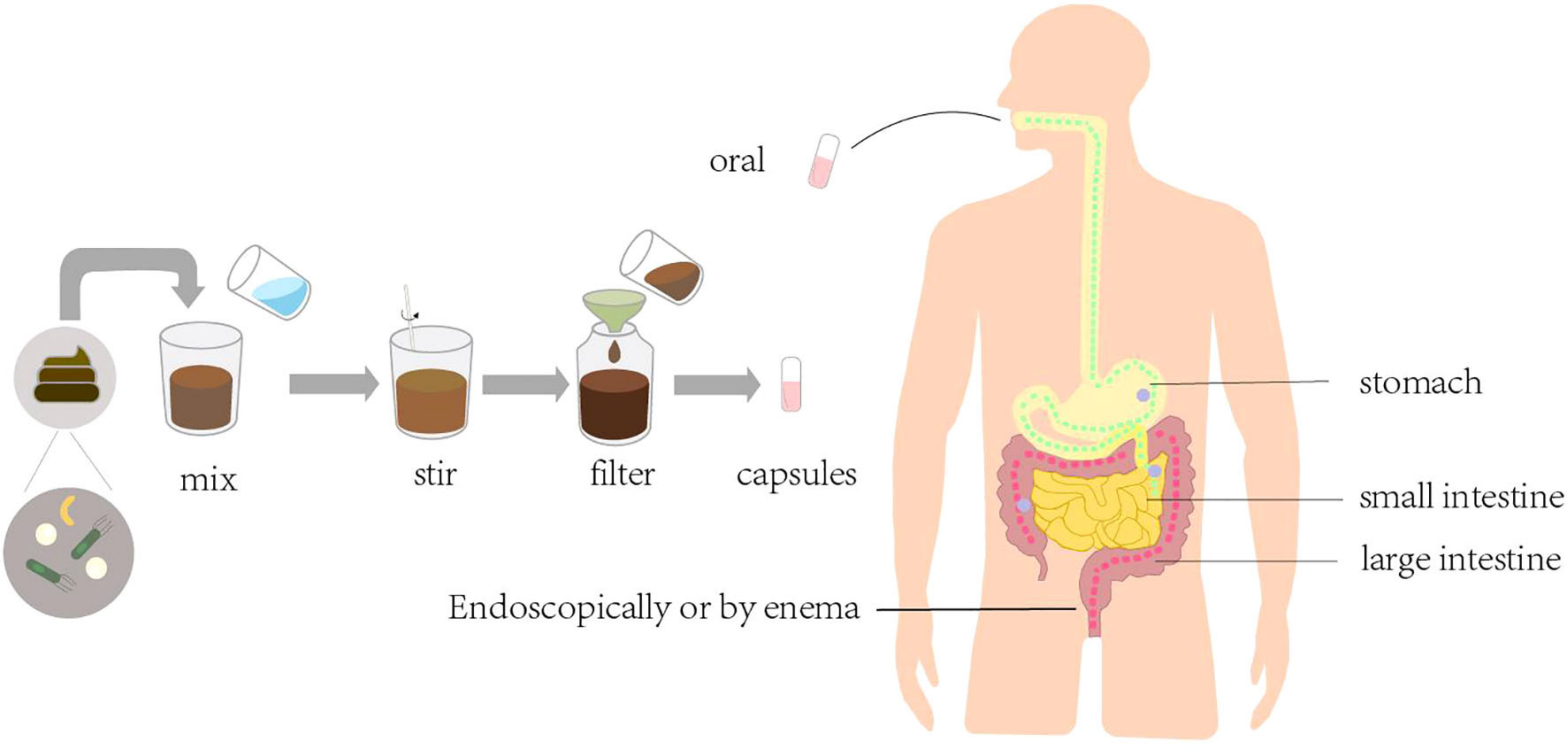
Evidence of association between microbial therapy and organ transplantation.

FMT is the transfer of feces from healthy donors to the colon of patients with diseases caused by altered microbiota, with the aim of restoring the normal microbiota to cure the disease (**Figure 3**). The earliest application of FMT was for the treatment of recurrent CDI (76). The first successful FMT procedure was performed in 2012 in an immunocompromised allogeneic stem cell transplant recipient with severe CDI (77). Subsequently, a multicenter, retrospective study demonstrated the safety and efficacy of FMT in the treatment of severe or explosive CDI after organ transplantation (70). In a 2016 case report, FMT was administered to patients with acute rejection after liver transplantation, and clinical symptoms improved and intestinal mucosal repair appeared within 24 hours after transplantation (78).

In addition, FMT can effectively improve microbial diversity (79), rebuild the composition of intestinal microbiota, and protect the graft from multi-drug-resistant bacterial damage (71). However, although FMT effectively prevents the development of CDI after transplantation by reestablishing microbiota complexity, it is still largely hindered by undefined microbiome-coding genes and gene clusters critical for resistance to CDI.

Bacteriophage therapy (BT) has been proposed as a new strategy to prevent multi-drug-resistant bacterial infections. Among the 3 lung transplant recipients with life-threatening multi-drug-resistant bacterial infections caused by *Pseudomonas aeruginosa* (n=2) and *Burkholderia dolosa* (n=1), 2 were successfully discharged from the hospital off ventilator support after BT treatment, and no adverse events related to BT were found in any of the 3 cases (72).

Microbial therapy has shown great potential in organ transplantation. These therapies reduce organ transplantation-related complications by regulating the intestinal microecology (**Table 4**). Despite their promising prospects, the safety, efficacy, and standardized application of these techniques still face many challenges. Further clinical studies are needed to validate and optimize them to promote their widespread application in organ transplantation.

**Table 4:** Evidence of the association between microbial therapy and organ transplantation.

Subject of study	Treatment method	Outcomes	Reference
Mice	Probiotic preparations containing Blautia products were administered by gavage.	Improve vancomycin-resistant enterococcal infections.	(66)
66 liver transplant patients	Oral or nasogastric compound probiotic preparations consisting of *Pediacoccus pentosaceus, Leuconostoc mesenteroides, Lactobacillus paracasei*, and *Lactobacillus plantarum*.	It can effectively reduce the incidence of bacterial nosocomial infection after transplantation.	(67)
55 liver transplant patients	4 strains of probiotic preparations	The infection rate was significantly reduced in 30 days and 90 days after operation, and the liver function index was also reduced.	(68)
56 kidney transplant patients	Oral prebiotic supplements	It reduced the infection rate and improved the gastrointestinal symptoms after transplantation	(69)
94 solid organ transplant patients	FMT	Clostridium difficile infection was effectively reduced.	(70)
10 patients with Clostridium difficile infection	FMT	Clostridium difficile was transplanted by increasing the abundance of Firmicutes and Bacteroidetes.	(79)
3 lung transplant patients infected with multidrug-resistant bacteria	BT	2 patients were discharged after significant improvement in clinical symptoms, and no treatment-related adverse events occurred.	(72)

## 6 Summary

In summary, IRI, as a key factor affecting the success of organ transplantation, not only directly damages the graft function but also leads to a series of postoperative complications by destroying the intestinal barrier and microecological balance. The intestinal microecology plays an important role in the recovery process after transplantation, and its imbalance is closely related to infection, acute rejection, and increased graft mortality. Therefore, maintaining and reconstructing the stability of the intestinal microecology is of great significance in reducing the incidence of complications after transplantation. Microbial therapies, such as probiotics and fecal microbial transplantation, have shown great potential in improving the transplant prognosis. Future studies should further reveal the mechanistic link between intestinal microbiota and organ transplantation and explore individualized microbial therapy strategies to optimize the effects of organ transplantation and improve the long-term prognosis of patients. In-depth study and regulation of the intestinal microecology will provide a new therapeutic perspective and method for organ transplantation.
